# A hypomorphic *BMPR1B* mutation causes du Pan acromesomelic dysplasia

**DOI:** 10.1186/s13023-015-0299-5

**Published:** 2015-06-24

**Authors:** Katja Stange, Julie Désir, Naseebullah Kakar, Thomas D. Mueller, Birgit S. Budde, Christopher T. Gordon, Denise Horn, Petra Seemann, Guntram Borck

**Affiliations:** Berlin-Brandenburg Center for Regenerative Therapies (BCRT), Charité Universitätsmedizin Berlin, 13353 Berlin, Germany; Berlin-Brandenburg School for Regenerative Therapies (BSRT), Charité-Universitätsmedizin Berlin, 13353 Berlin, Germany; Institut de Pathologie et de Génétique, 6041 Gosselies, Belgium; Institute of Human Genetics, University of Ulm, 89081 Ulm, Germany; Julius-von-Sachs Institute, University of Würzburg, 97082 Würzburg, Germany; Cologne Center for Genomics (CCG), University of Cologne, Cologne, Germany; INSERM UMR 1163, Institut Imagine, Paris, 75015 France; Université Paris Descartes-Sorbonne Paris Cité, Institut Imagine, Paris, 75015 France; Institute for Medical and Human Genetics, Charité-Universitätsmedizin Berlin, 13353 Berlin, Germany

**Keywords:** Acromesomelic dysplasias, Grebe dysplasia, du Pan dysplasia, *BMPR1B*

## Abstract

**Background:**

Grebe dysplasia, Hunter-Thompson dysplasia, and du Pan dysplasia constitute a spectrum of skeletal dysplasias inherited as an autosomal recessive trait characterized by short stature, severe acromesomelic shortening of the limbs, and normal axial skeleton. The majority of patients with these disorders have biallelic loss-of-function mutations of *GDF5*. In single instances, Grebe dysplasia and a Grebe dysplasia-like phenotype with genital anomalies have been shown to be caused by mutations in *BMPR1B*, encoding a GDF5 receptor.

**Methods:**

We clinically and radiologically characterised an acromesomelic chondrodysplasia in an adult woman born to consanguineous parents. We sequenced *GDF5* and *BMPR1B* on DNA of the proposita. We performed 3D structural analysis and luciferase reporter assays to functionally investigate the identified *BMPR1B* mutation.

**Results:**

We extend the genotype-phenotype correlation in the acromesomelic chondrodysplasias by showing that the milder du Pan dysplasia can be caused by a hypomorphic *BMPR1B* mutation. We show that the homozygous c.91C>T, p.(Arg31Cys) mutation causing du Pan dysplasia leads to a significant loss of BMPR1B function, but to a lesser extent than the previously reported p.Cys53Arg mutation that results in the more severe Grebe dysplasia.

**Conclusions:**

The phenotypic severity gradient of the clinically and radiologically related acromesomelic chondrodysplasia spectrum of skeletal disorders may be due to the extent of functional impairment of the ligand-receptor pair GDF5-BMPR1B.

## Background

The acromesomelic dysplasias (ACD) constitute a rare subgroup of osteochondrodysplasias that are characterised by short stature and shortened limbs with anomalies of the hands and feet [1]. According to the 2010 revision of the nosology and classification of genetic skeletal disorders, five ACD subgroups are recognised, namely the severe Grebe dysplasia (including Grebe (OMIM #200700) and Hunter-Thompson (OMIM #201250) types), the milder du Pan dysplasia (OMIM #228900), a Grebe dysplasia-like phenotype with genital anomalies (OMIM #609441), as well as the ACD types Maroteaux (OMIM #602875) and Osebold-Remondini (OMIM 112910) (the latter two types will not be considered further here) [2]. While Grebe dysplasia is characterised by severe distal limb anomalies with rudimentary fingers and toes, there is mild short stature and milder limb involvement in du Pan dysplasia, including fibular hypoplasia and complex brachydactyly.

Both Grebe dysplasia and du Pan dysplasia are autosomal recessive disorders and can be caused by biallelic loss-of-function mutations of *GDF5* (previously known as *CDMP1*), encoding the growth and differentiation factor 5 [3–6]. GDF5 belongs to the bone morphogenetic protein (BMP) family and binds to the BMP receptors BMPR1A and BMPR1B, with a preference for BMPR1B [7]. Consistent with this ligand-receptor interaction, ACD cannot only be caused by *GDF5* mutations, but in rare cases also by loss-of-function *BMPR1B* mutations. Indeed, Demirhan and colleagues identified a homozygous truncating mutation of *BMPR1B* underlying a Grebe dysplasia-like phenotype with genital anomalies (absent ovaries, hypoplastic uterus, hypergonadotropic hypogonadism and primary amenorrhea) [8]; and we previously reported a homozygous missense mutation (p.Cys53Arg) and a homozygous nonsense mutation (p.Trp219*) of *BMPR1B* in Grebe dysplasia [9]. Functional analysis of the p.Cys53Arg missense alteration indicated that the mutant receptor had reduced localisation at the cell membrane, reduced activation by GDF5 binding and no effect on cell differentiation upon overexpression in an *in vitro* chondrogenesis assay, consistent with loss of function [9]. Here we have identified and characterised a hypomorphic *BMPR1B* missense alteration that causes du Pan dysplasia.

## Methods

### Clinical investigation and molecular analyses

The clinical evaluation was performed at the Department of Medical Genetics, Hospital Erasme, Brussels, Belgium, and the Institut de Pathologie et de Génétique, Gosselies, Belgium, and research was performed following a protocol approved by the University of Ulm Ethics Committee. After obtaining written informed consent for participation in the study, including consent to report individual patient data, we sequenced the coding exons and splice sites of *GDF5* (NM_000557.2) and *BMPR1B* (NM_001256794.1) on genomic DNA after PCR amplification. Primer sequences are available on request. PCR products were sequenced on an ABI 3730 DNA Analyzer with BigDye chemistry v3.1 (Applied Biosystems). Sequence traces were assembled, aligned, and analyzed with the Seqman software (DNASTAR Lasergene). The *BMPR1B* intragenic short tandem repeat (STR) markers D4S2278 and *BMPR1B-STR2* (forward primer 5’-TTAAAAGTAAAGACCATATAAAGG-3’; reverse primer 5’-GGACTTAAAACTCTGCAACAAC-3’) were genotyped after PCR amplification on an ABI 3730 DNA Analyzer. Single nucleotide polymorphism (SNP) array genotyping was performed using a Genome-Wide Human SNP Array 6.0 (Affymetrix), according to the manufacturer´s instructions. Linkage analysis and haplotype reconstruction were performed using the program ALLEGRO [10]. Haplotypes were presented graphically with HaploPainter [11]. All data handling was performed using the graphical user interface ALOHOMORA [12].

### Structural analysis

The BMPR1B substitutions Arg31Cys (identified here) and Arg31His (present at low frequency in public databases; rs200035802) were modeled on the basis of the crystal structure of the human BMPR1B extracellular domain bound to human GDF5 (PDB entry 3EVS, [13]). The side chain of Arg31 was exchanged for either a cysteine or histidine using the ProteinDesign tool of the software Quanta2008 (MSI Accelrys) and rotamer searches were performed to identify the most likely side chain conformation. Potential alternative disulfide connectivities were analyzed by performing energy minimization runs *in vacuo* employing only geometrical energy terms.

### Cloning of BMPR1B expression constructs

The expression constructs for HA-tagged *Bmpr1b* WT and *Bmpr1b* Cys53Arg were previously described [9]. HA-tagged *Bmpr1b* WT in pSLAX13 was used as a template for *in vitro* mutagenesis to introduce the mutation coding for Arg31Cys (Primer forward 5´-ctcggcccaagatcctaTgttgtaaatgccaccac-3´, reverse 5´-gtggtggcatttacaacAtaggatcttgggccgag-3´) or coding for Arg31His (Primer forward 5´-cggcccaagatcctacAttgtaaatgccaccac-3´, reverse 5´-gtggtggcatttacaaTgtaggatcttgggccg-3´). Inserts were subcloned into the expression vector pCS2+ after ClaI restriction.

### Luciferase reporter gene assay

NIH/3 T3 cells (ATCC) were seeded in a 96-well plate in growth medium (DMEM 4.5 g/l glucose (Lonza), 10 % FBS superior (Biochrom), 2 mM L-glutamine (Lonza)) and transfected 24 h later using Lipofectamine 2000 (Invitrogen, Life Technologies) following the manufacturer´s instructions. Cells were transfected in growth medium with the control vector pCS2+ or one of the *Bmpr1b* variants in pCS2+ together with the BRE luciferase reporter construct BRE-pLG3ti [14] and the Renilla luciferase normalization vector pRL-TK (Promega). After 18 h cells were stimulated with 2 nM human recombinant GDF5 (Biopharm) in serum-reduced medium (DMEM 4.5 g/l glucose (Lonza), 1 % FBS superior (Biochrom), 2 mM L-glutamine (Lonza)). 40 h after transfection cells were lysed in potassium phosphate buffer (9 mM potassium di-hydrogen phosphate, 91 mM di-potassium phosphate, 0.2 % Triton-X-100) and dual luciferase activity was measured as described previously [15] using the Mithras LB 940 (Berthold Technologies). For statistical analysis GraphPad Prism 5 (GraphPad Software, Inc.) was used.

### Confocal microscopy

NIH/3 T3 cells were seeded into a 24-well plate in growth medium on cover glasses (Marienfeld). 24 h later *Bmpr1b* expression vectors were transfected into the NIH/3 T3 cells using Lipofectamine 2000 (Invitrogen, Life Technologies) following the manufacturer´s instructions. Another 24 h later cells were incubated under serum free conditions (DMEM 4.5 g/l glucose (Lonza), 2 mM L-glutamine (Lonza)) for 1 h, subsequently fixed with 4 % paraformaldehyde in PBS and blocked in PBS containing 10 % FBS superior (Biochrom) over night. Non-permeated cells were incubated with a rabbit anti-HA antibody (H6908, Sigma-Aldrich; diluted 1:100 in PBS containing 10 % FBS superior (Biochrom)) for 1 h. After washing with PBS the cells were incubated with the secondary antibody anti-rabbit-Alexa Fluor 488 (A11008, Molecular Probes Life Technologies) and with DAPI (Invitrogen, Life Technologies) for 1 h. The cover glasses containing stained cells were mounted on microscope slides (‘SuperFrost Plus’, Menzel) using Fluoromount-G (Southern Biotech). Confocal microscopy was performed using Zeiss Axio Imager.M2 equipped with a LSM700 confocal module (Carl Zeiss, 63-fold magnification).

## Results

### Clinical evaluation

**Fig. 1:**
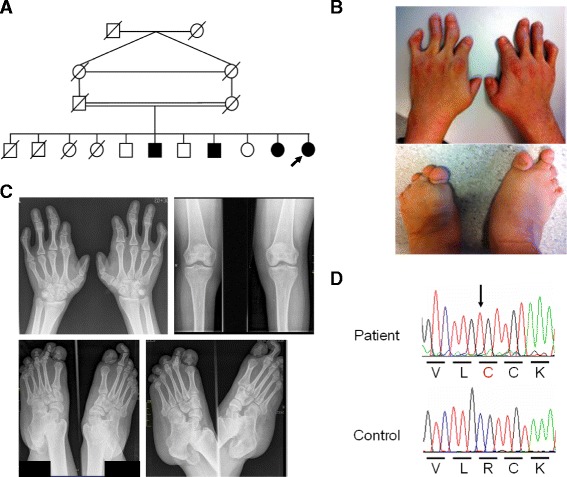
BMPR1B p.Arg31Cys causes a du Pan dysplasia-like phenotype. **a** Pedigree of the family. The proposita is indicated by an arrow. **b** Hands and feet of the proposita. Note short, malformed fingers and hypoplastic toes. **c** Radiographs of the proposita. Ap view of hands at the age of 32 years showing abnormalities of metacarpals and phalangeal bones. Ap view of the knees: note absent fibulae. Radiographs of the lower extremities including feet; ap and lateral view showing abnormalities of tarsal, metatarsal and phalangeal bones. **d** Sequence chromatograms showing parts of *BMPR1B* exon 1. The homozygous c.91C>T, p.(Arg31Cys) mutation is indicated by an arrow. Letters below the sequence chromatograms indicate amino acids in single letter code

The proposita was a 38 year-old woman who presented to the genetics clinic because of short stature and limb anomalies. She is the 11^th^ child (of 11) born to first cousin parents originating from Morocco (Fig. 1a). One of her sisters and two of her brothers are similarly affected; they live in a remote area of Morocco with very limited access to medical care and no detailed clinical information, skeletal radiographs or DNA were available from them. The parents were unaffected; maternal height was 160 cm and paternal height 180 cm.

The proposita had disproportionate short stature with a height of 148 cm (− 2.7 SD) and mild acromesomelic limb shortening. The fibulae were not palpable. She had short fingers and abnormal finger joints with deviations (Fig. 1b). Fingers II and V were most severely affected. Finger nails were normal. The toes were hypoplastic, in particular toes III to V, with broad and short toe nails (Fig. 1b). Radiological examination for the hands revealed proximal symphalangism and dysplastic middle phalanges (absence of the middle phalanx in the index finger, hypoplasia of the middle phalanges with narrow proximal phalangeal joints in the middle and ring fingers, and absence of the middle phalanx and long proximal phalanx in the little finger), shortening of the first metacarpal and a single long phalanx in the thumb, short proximal phalanx of the index finger and narrow carpal joint spaces (Fig. 1c). Radiographs of the feet showed similar findings, including a single misshapen phalanx in the great toe, hypoplasia of the middle phalanx in the second toe, absence of the middle phalanges in the third, fourth, and fifth toes, and calcaneocuboid fusion (Fig. 1c). The ankle joints were mal-aligned. The fibula was totally missing (Fig. 1c).

The proposita reported normal puberty with menarche at 13 years and regular menstrual cycles. Abdominal ultrasound was normal with the presence of a uterus and ovaries of normal size and shape. There was no hypergonadotropic hypogonadism with normal LH, FSH, estradiol and progesterone.

### Mutation identification

Because the clinical presentation was compatible with du Pan dysplasia we first sequenced the coding region and exon-intron boundaries of *GDF5* and identified no mutation. Upon sequencing of *BMPR1B* we detected a homozygous c.91C>T (p.Arg31Cys) variant (Fig. 1d). No DNA from other affected and unaffected family members was available to test for co-segregation with du Pan dysplasia. Consistent with *BMPR1B* being located in an autozygous region, genotyping of two highly polymorphic *BMPR1B* intragenic STRs showed homozygosity and SNP array genotyping confirmed a large (20.2 Mb) homozygous region at the *BMPR1B* locus in the proposita, defined by the flanking SNPs rs2131361 and rs17278473 (data not shown). Residue Arg31 is moderately conserved in BMPR1B orthologs. It is conserved e.g., in mouse, rat, cat, dog, and platypus; it is replaced with a histidine in e.g., green monkey, panda and hedgehog, and with a glutamine in e.g., opossum and turtles. None of the >60 species included in the UCSC multiple species alignment has a cysteine at the corresponding position. While the ExAC browser (http://exac.broadinstitute.org/) lists a p.Arg31His substitution (rs200035802) with an allele frequency of 18/121.162, p.Arg31Cys was only identified in 1/121.150 alleles, compatible with p.Arg31Cys being disease causing. Consistently, PolyPhen-2 [16] predicts p.Arg31Cys as “possibly damaging” and p.Arg31His as “benign” (both variants are predicted “not tolerated” by SIFT [17]). Finally, no variant predicting p.Arg31Cys was identified in exome sequences from 30 Moroccan patients with unrelated disorders (Jaber Lyahyai, personal communication) and no p.Arg31Cys homozygotes or heterozygotes were present in the 5,718 exomes included in the Institut Imagine (Paris) in-house database, which is enriched for exomes of Maghrebian individuals, and contains a very conservatively estimated number of Moroccan exomes of 33.

### Structural modeling of the BMPR1B mutation p.Arg31Cys

**Fig. 2:**
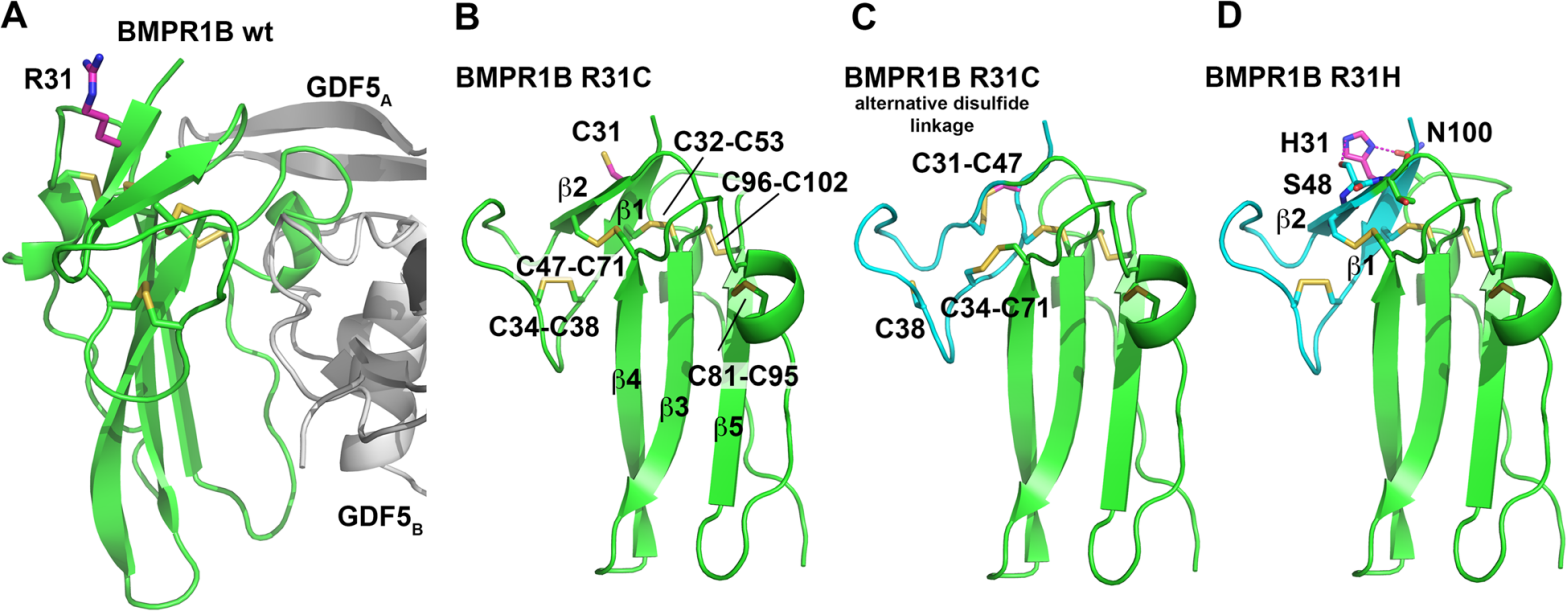
3D models for Arg31 substitutions in human BMPR1B. **a** Structure of the extracellular domain of BMPR1B (*green*) bound to GDF5 (*grey*). Position 31 is indicated with the C-atoms colored in magenta. **b** Model of the BMPR1B variant Arg31Cys. The unpaired cysteine residue located in β-strand 1 is highlighted in magenta; the five disulfide bonds formed in BMPR1B WT are shown as *yellow* sticks and are marked accordingly. **c** As in (**b**) but with an alternative disulfide bond network. The close proximity of the introduced cysteine residue at position 31 to the native residues Cys32, Cys47, Cys53, and Cys71 potentially leads to an alternative connectivity, which could then alter the conformation of the loops. If Cys31 connects to Cys47 an alternative non-native disulfide bond between Cys34 and Cys71 might be formed, which alters the conformation of the β1β2-loop important in the recognition and binding of BMP ligands. **d** Model of the BMPR1B variant Arg31His. The shorter histidine side chain places the hydrogen bond donor and acceptor groups of the imidazole ring in close proximity to Ser48 and Asn100 allowing to form new hydrogen bonds with the latter two residues thereby possibly altering the conformation of β-strand 2 and the β1β2-loop

Arg31 is located at the beginning of the BMPR1B ligand binding domain (LBD), suggesting that the p.Arg31Cys mutation may interfere with GDF5 binding. We modeled p.Arg31Cys using the structure of human BMPR1B bound to human GDF5 [13]. Residue 31 shares no direct contact with the ligand GDF5 and is more than 10 Å remote (Fig. 2a). Thus, a direct impact on the GDF5-receptor binding by loss of direct van der Waals interactions seems unlikely. In our *in silico* models the exchange of Arg31 by cysteine however places an unpaired thiol group in close proximity to Cys32, Cys47, Cys53, and Cys71 (Fig. 2b) thereby possibly allowing for an alternate disulfide network. The model of such a rearranged disulfide bond network suggested that it will not necessarily destroy the BMP receptor’s three-finger toxin fold, but may introduce conformational changes in the disulfide-bond connected loops in particular of the β1β2-loop (Fig. 2c). As this loop is engaged in contacts with GDF5 and has been implicated in ligand recognition and binding (Fig. 2a) [13] even such smaller changes may affect ligand-receptor interaction. The effects of p.Arg31His seem subtler as those predicted for p.Arg31Cys (Fig. 2d).

### Functional analyses of mutant BMPR1B receptors

**Fig. 3:**
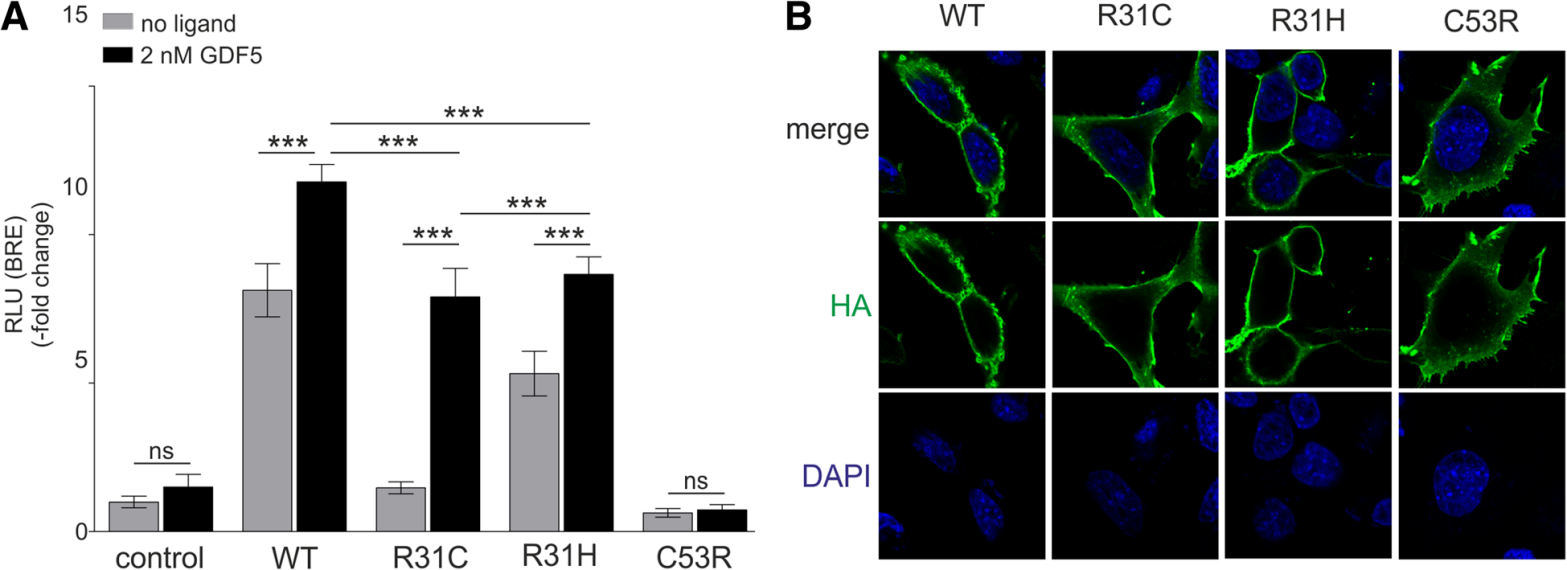
The Arg31Cys substitution in BMPR1B causes a moderate loss of function. **a** NIH/3 T3 cells were co-transfected with the empty vector pCS2+ or the indicated HA-tagged variants of *Bmpr1b* with the luciferase constructs BRE-pLG3ti and pRL-TK and stimulated with 2 nM of human recombinant GDF5. BMPR1B WT strongly induced luciferase activity. The variant BMPR1B Arg31Cys showed nearly no activity without ligand stimulation. It was activated after GDF5 treatment but could not accomplish the level of BMPR1B WT. Arg31His induced signaling, but considerably lower compared to BMPR1B WT. BMPR1B Cys53Arg did not activate SMAD signaling at all. Data were tested for normal distribution (Kolmogorov-Smirnoff normality test) and analyzed using a One-Way-ANOVA with subsequent Bonferroni's Multiple Comparison Test (n = 8; ns not significant, *** *p* < 0.001). **b** NIH/3 T3 cells were transfected with the indicated HA-tagged *Bmpr1b* variants. Using an anti-HA antibody the expression of BMPR1B could by visualized via confocal microscopy. DAPI was used for staining of cell nuclei. All BMPR1B variants were translocated to the cell membrane

We performed a luciferase reporter gene assay using the SMAD dependent BRE reporter to determine the signal transduction capacity of the BMPR1B variant Arg31Cys, which is associated with ACD du Pan type in our patient (Fig. 3a). We compared its activity to the previously described BMPR1B mutation Cys53Arg, which is associated with ACD Grebe type and to Arg31His, another allele listed in genomic databases [9]. The WT receptor induced the luciferase expression approximately 8-fold compared to a control vector and was strongly stimulated by the presence of recombinant GDF5. BMPR1B Arg31Cys showed only moderate activity when no ligand was used for stimulation (less than 2-fold). Arg31His induced the SMAD signaling pathway approximately 5-fold, but remained significantly less active than the WT receptor. SMAD1/5/8 activation via the BMPR1B variants Arg31His and Arg31Cys was significantly increased upon ligand stimulation. However, BMPR1B Arg31Cys still could not accomplish the level of BMPR1B Arg31His or BMPR1B WT, pointing to a partial loss of function. In line with previous findings BMPR1B Cys53Arg exhibited no SMAD activity and could not be stimulated by GDF5 either. Confocal microscopy showed that all investigated BMPR1B variants are translocated to the cell surface (Fig. 3b).

## Discussion

We have identified the homozygous *BMPR1B* missense mutation p.Arg31Cys in a patient with ACD du Pan type. Previously, *GDF5* mutations have been shown to constitute the major cause of ACD Grebe and du Pan types [3–6], and *BMPR1B* mutations had been identified in single families with Grebe syndrome and a Grebe dysplasia-like phenotype with genital anomalies [8, 9]. However, whether *BMPR1B* mutations, presumably mutations with milder functional effects, can also cause du Pan dysplasia was unknown. The following evidence supports a diagnosis of du Pan dysplasia in the proposita rather than another ACD or ACD-like skeletal dysplasia: normal appearing radius, ulna and femur including the corresponding joints; mal-shaped but present phalangeal bones of the hands; normal length of the second metacarpal; and milder involvement of the tarsals, metatarsals, and phalangeal bones of the toes. In Grebe and Hunter-Thompson ACD the clinical and radiological manifestations are more severe: the humerus, radius and ulna are short and bowed, the hand bones are more deformed or missing and the elbow and knee joints are affected. Thus, our results show that both Grebe and du Pan dysplasia can be caused by *BMPR1B* mutations in rare cases. While the number of affected individuals with ACD due to *BMPR1B* mutations is small, our previous [9] and present results indicate a possible genotype-phenotype correlation according to which *BMPR1B* mutations with a strong functional effect would cause Grebe syndrome, while milder mutations would result in the clinically and radiologically milder du Pan dysplasia.

Indeed, when the Arg31Cys mutation or the Arg31His variant are compared with other *BMPR1B* mutations, e.g., the Cys53Arg exchange [9], the assumed impact of a missense substitution at position 31 on structure and BMP binding seems rather moderate. The substitution of Cys53 not only disrupts the structure-stabilizing disulfide bond network, but also likely causes local unfolding in the center of the BMPR1B ligand-binding domain due to strong van der Waals overlaps from introducing a bulky, charged arginine side chain into the tightly packed hydrophobic core around Cys53. In contrast, the side chain of Arg31 faces the protein surface being surrounded by bulk solvent. Thus, exchanges against most other amino acid types will likely not alter the structure of the BMPR1B ligand-binding domain. Furthermore the residue at position 31 does not contact GDF5, thus only amino acid substitutions that (indirectly) cause a conformational rearrangement of ligand-binding loops can affect BMP binding.

A luciferase reporter gene assay indeed revealed that both BMPR1B variants, Arg31Cys and Arg31His, can still be stimulated by GDF5 but signal with diminished biological activity compared to BMPR1B WT. This suggests that the exchanges at position Arg31 have for that matter only moderate functional effect and do not completely prevent ligand binding, as it is the case for Cys53Arg.

## Conclusion

In conclusion, we provide evidence for a causal role of a hypomorphic BMPR1B missense mutation in du Pan dysplasia, thereby broadening the clinical and molecular overlap between ACD types. This finding also has obvious implications for molecular diagnostic strategies, as *BMPR1B* sequencing should be considered in patients with ACD harboring no mutations in *GDF5*.

## References

[1] Krakow D, Rimoin DL (2010). The skeletal dysplasias. Genet Med.

[2] Warman ML, Cormier-Daire V, Hall C, Krakow D, Lachman R, LeMerrer M (2011). Nosology and classification of genetic skeletal disorders: 2010 revision. Am J Med Genet A.

[3] Thomas JT, Kilpatrick MW, Lin K, Erlacher L, Lembessis P, Costa T (1997). Disruption of human limb morphogenesis by a dominant negative mutation in CDMP1. Nat Genet.

[4] Faiyaz-Ul-Haque M, Ahmad W, Zaidi SH, Haque S, Teebi AS, Ahmad M (2002). Mutation in the cartilage-derived morphogenetic protein-1 (CDMP1) gene in a kindred affected with fibular hypoplasia and complex brachydactyly (DuPan syndrome). Clin Genet.

[5] Faiyaz-Ul-Haque M, Ahmad W, Wahab A, Haque S, Azim AC, Zaidi SH (2002). Frameshift mutation in the cartilage-derived morphogenetic protein 1 (CDMP1) gene and severe acromesomelic chondrodysplasia resembling Grebe-type chondrodysplasia. Am J Med Genet.

[6] Stelzer C, Winterpacht A, Spranger J, Zabel B (2003). Grebe dysplasia and the spectrum of CDMP1 mutations. Pediatr Pathol Mol Med.

[7] Nickel J, Kotzsch A, Sebald W, Mueller TD (2005). A single residue of GDF-5 defines binding specificity to BMP receptor IB. J Mol Biol.

[8] Demirhan O, Turkmen S, Schwabe GC, Soyupak S, Akgul E, Tastemir D (2005). A homozygous BMPR1B mutation causes a new subtype of acromesomelic chondrodysplasia with genital anomalies. J Med Genet.

[9] Graul-Neumann LM, Deichsel A, Wille U, Kakar N, Koll R, Bassir C (2014). Homozygous missense and nonsense mutations in BMPR1B cause acromesomelic chondrodysplasia-type Grebe. Eur J Hum Genet.

[10] Gudbjartsson DF, Jonasson K, Frigge ML, Kong A (2000). Allegro, a new computer program for multipoint linkage analysis. Nat Genet.

[11] Thiele H, Nürnberg P (2005). HaploPainter: a tool for drawing pedigrees with complex haplotypes. Bioinformatics.

[12] Rüschendorf F, Nürnberg P (2005). ALOHOMORA: a tool for linkage analysis using 10 K SNP array data. Bioinformatics.

[13] Kotzsch A, Nickel J, Seher A, Sebald W, Muller TD (2009). Crystal structure analysis reveals a spring-loaded latch as molecular mechanism for GDF-5-type I receptor specificity. EMBO J.

[14] Korchynskyi O, ten Dijke P (2002). Identification and functional characterization of distinct critically important bone morphogenetic protein-specific response elements in the Id1 promoter. J Biol Chem.

[15] Hampf M, Gossen M (2006). A protocol for combined Photinus and Renilla luciferase quantification compatible with protein assays. Anal Biochem.

[16] Adzhubei IA, Schmidt S, Peshkin L, Ramensky VE, Gerasimova A, Bork P (2010). A method and server for predicting damaging missense mutations. Nat Methods.

[17] Kumar P, Henikoff S, Ng PC (2009). Predicting the effects of coding non-synonymous variants on protein function using the SIFT algorithm. Nat Protoc.

